# HIF-1α triggers ER stress and CHOP-mediated apoptosis in alveolar epithelial cells, a key event in pulmonary fibrosis

**DOI:** 10.1038/s41598-018-36063-2

**Published:** 2018-12-18

**Authors:** Eva Delbrel, Abdoulaye Soumare, Adnan Naguez, Rabab Label, Olivier Bernard, Alain Bruhat, Pierre Fafournoux, Geoffrey Tremblais, Dominique Marchant, Thomas Gille, Jean-François Bernaudin, Patrice Callard, Marianne Kambouchner, Emmanuel Martinod, Dominique Valeyre, Yurdagül Uzunhan, Carole Planès, Emilie Boncoeur

**Affiliations:** 1 Université Paris 13, Sorbonne Paris Cité, Laboratoire ‘Hypoxie & Poumon’ (EA 2363), F-93017 Bobigny, France; 2 APHP, Hôpital Avicenne, F-93017 Bobigny, France; 3 Institut National de la Recherche Agronomique (INRA), UMR-1019 Nutrition Humaine, Centre INRA Auvergne Rhône-Alpes, Clermont Auvergne Université, 63122 Saint Genès Champanelle, France; 4 Sorbonne Université, Faculté de Médecine, 75013 Paris, France

## Abstract

Endoplasmic Reticulum (ER) stress of alveolar epithelial cells (AECs) is recognized as a key event of cell dysfunction in pulmonary fibrosis (PF). However, the mechanisms leading to AECs ER stress and ensuing unfolded protein response (UPR) pathways in idiopathic PF (IPF) remain unclear. We hypothesized that alveolar hypoxic microenvironment would generate ER stress and AECs apoptosis through the hypoxia-inducible factor-1α (HIF-1α). Combining *ex vivo*, *in vivo* and *in vitro* experiments, we investigated the effects of hypoxia on the UPR pathways and ER stress-mediated apoptosis, and consecutively the mechanisms linking hypoxia, HIF-1α, UPR and apoptosis. HIF-1α and the pro-apoptotic ER stress marker C/EBP homologous protein (CHOP) were co-expressed in hyperplastic AECs from bleomycin-treated mice and IPF lungs, not in controls. Hypoxic exposure of rat lungs or primary rat AECs induced HIF-1α, CHOP and apoptosis markers expression. In primary AECs, hypoxia activated UPR pathways. Pharmacological ER stress inhibitors and pharmacological inhibition or silencing of HIF-1α both prevented hypoxia-induced upregulation of CHOP and apoptosis. Interestingly, overexpression of HIF-1α in normoxic AECs increased UPR pathways transcription factors activities, and CHOP expression. These results indicate that hypoxia and HIF-1α can trigger ER stress and CHOP-mediated apoptosis in AECs, suggesting their potential contribution to the development of IPF.

## Introduction

Idiopathic pulmonary fibrosis (IPF), the most common and severe form of interstitial lung diseases, is pathologically characterized by a pattern of usual interstitial pneumonia (UIP) associating fibrotic remodelling leading to honeycombing and abnormal characteristics of the alveolar epithelial cells (AECs)^1^. IPF is thought to be the consequence of repetitive micro-injuries of the alveolar epithelium, followed by inefficient repair and uncontrolled activation and proliferation of (myo) fibroblasts^2,3^. Alveolar type II (ATII) cells, instead of proliferating to recover the denuded basal membrane, undergo apoptosis or a transformation through epithelial-mesenchymal transition (EMT)^4^. In addition, some AECs turn into hyperplastic cells with abnormal activation and production of pro-fibrotic factors^5^.

Interestingly, endoplasmic reticulum (ER) stress markers have been evidenced in AECs from patients with IPF, suggesting a potential role for ER stress in the pathogenesis of the disease^6,7^. To compensate cell damage and disturbed folding of proteins in the ER, the Unfolded Protein Response (UPR) signaling is induced. The three branches of UPR, ATF4, spliced X-box binding protein 1 (XBP1s) and ATF6α transcription factors are activated to inhibit protein translation, activate ER chaperone transcription and ER Activation Degradation (ERAD)^8^. If ER dysfunction is severe or prolonged, the UPR activation can result in fibrotic remodelling through induction of EMT or activation of pro-apoptotic pathways^9,10^ in part *via* the induction of the pro-apoptotic transcription factor C/EBP homologous protein (CHOP)^11^ and regulation of its target genes *Bcl2*, *Bim* and *Chac-1*^12–14^.

While in some familial forms of pulmonary fibrosis the induction of ER stress could likely be related to the accumulation of misfolded mutated surfactant proteins within the ER^15,16^, the trigger(s) of ER stress induction in sporadic IPF is (are) still unknown. Considering the fact that hypoxia may promote ER stress in various organs^17,18^, and that the expression of the hypoxia-inducible factor 1α (HIF-1α) has been previously reported in AECs from IPF lungs^19^, we hypothesized that localized alveolar hypoxia and HIF-1α could be relevant stressors inducing prolonged ER stress and subsequent apoptosis of AECs in sporadic IPF.

Therefore, the objectives of the present study were: (1) to determine whether HIF-1α and CHOP proteins were co-expressed by AECs in lung tissue from IPF patients; (2) to evaluate whether micro-environmental hypoxia could activate the UPR pathways in rat AECs; (3) to decipher the molecular mechanisms linking hypoxia, HIF-1α, ER stress and apoptosis in these cells. Our results show that HIF-1α and CHOP proteins were both detected in hyperplastic AECs observed in IPF patients’ lung biopsies and in bleomycin-induced pulmonary fibrosis in mice as well as in AECs from rat exposed to acute hypoxia. *In vitro*, hypoxia-induced apoptosis was prevented by treatment with ER stress inhibitors salubrinal (SLB) and 4-phenylbutyrate (4-PBA) and by CHOP silencing. Finally, HIF-1α involvement in the regulation of the transcriptional capacity of ATF4 and ATF6α/XBP1s on their specific responsive elements, and CHOP expression was shown. Taken together, these results suggest that localized hypoxia of the alveolar milieu and expression of HIF-1α could promote UPR pathways, CHOP expression and apoptosis in AECs, therefore contributing to alveolar cell dysfunction and finally promoting lung fibrosis.

## Results

### HIF-1α and CHOP are expressed in alveolar epithelial cells from bleomycin-treated mice and in human IPF lung biopsies

**Figure 1:**
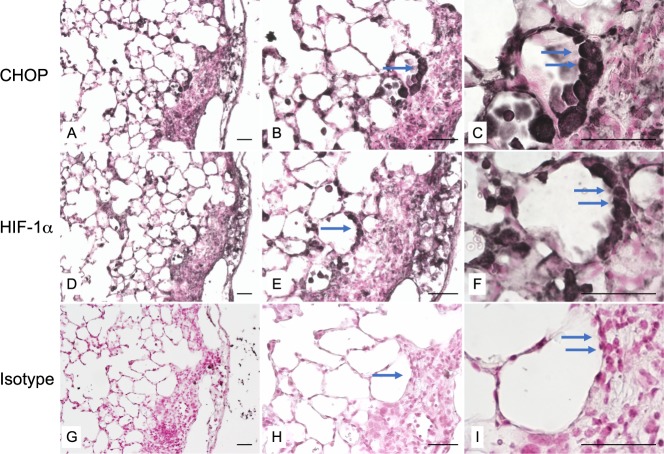
Coexpression of CHOP and HIF-1α in alveolar epithelial cells of bleomycin-induced lung fibrosis in mice. CHOP (**A**–**C**) and HIF-1α (**D**–**F**) are co-expressed (serial sections) in hyperplastic alveolar epithelial cells (AECs) in and close to areas of pulmonary remodelling (arrow). No labelling was observed with isotypic controls (**G**–**I**). Sections were counterstained with Nuclear Fast Red. BC are higher magnifications of A; EF of D and HI of G; Original magnification: objective X200 (**A**,**D**,**G**), x 400 (**B**,**E**,**H**) and x 1000 (**C**,**F**,**I**). Scale bars represent 50 μm.

**Figure 2:**
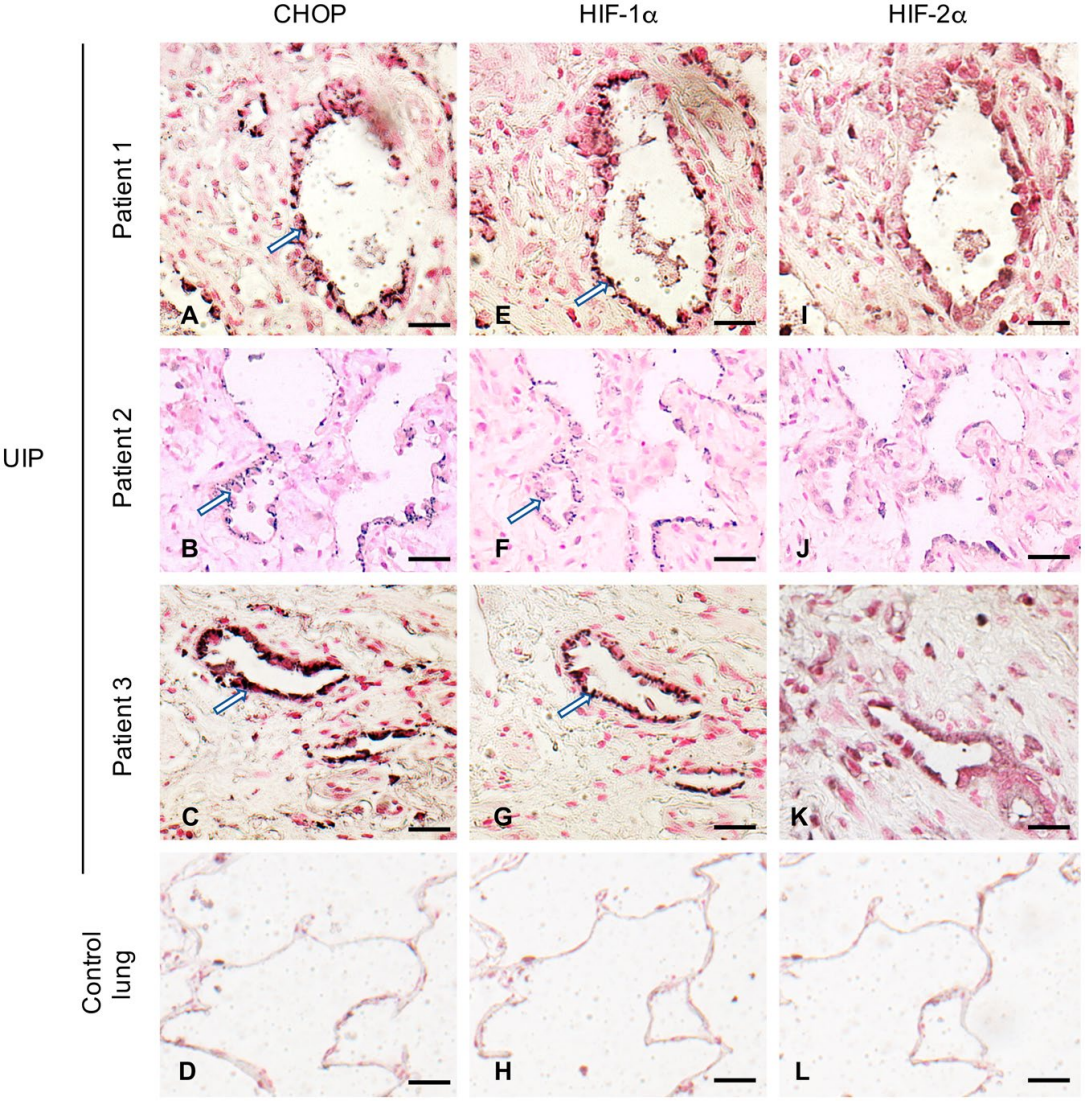
Co-expression of CHOP and HIF-1α in alveolar epithelial cells of patients with IPF. CHOP and HIF-1α are co-expressed in hyperplastic alveolar epithelial cells (arrow) in the UIP areas from 3 patients with IPF (UIP) (**A**–**C**,**E**–**G**). Expression of CHOP (**A**–**C**) and HIF-1α (**E**–**G**) in the same epithelial cells from serial sections of IPF lungs presenting a fibrotic pattern; no labelling for HIF-2α was observed (**I**–**K**). No labelling for CHOP (**D**), HIF-1α (**H**) or HIF-2α (**L**) was detected in normal control lung. Sections were counterstained with Nuclear Fast Red. Original magnification: objective X400. Scale bars represent 100 μm.

Immunostainings on serial sections of mouse lungs analyzed 21 days after bleomycin intratracheal instillation showed that both HIF-1α and CHOP were expressed in AECs in and close to characteristic areas of alveolar and interstitial remodeling (Fig. 1A–F). No labelling was observed in the lung of control mice (data not shown). Analysis of lung biopsies from 3 IPF patients showed a characteristic UIP pattern associating fibrotic remodelling with modified epithelial cells covering the parenchymal air spaces (Fig. 2A–C,E–G,I–K). Expression patterns of HIF-1α and CHOP were studied by immunostaining of serial sections of these biopsies and of control lung samples. HIF-1α and CHOP were co-expressed in hyperplastic AECs located in fibrotic areas displaying a typical UIP pattern (Fig. 2A–C,E–G,I–K). More precisely, at higher magnification, a supranuclear localization of both HIF-1α and CHOP was observed in these cells (Supplemental Fig. 1A,B,E,F). A more diffuse cytoplasmic distribution was also observed in reactive AECs in less fibrotic regions (Supplemental Fig. 1C,G). No significant labelling was observed for HIF-2α in fibrotic areas (Fig. 1I–K). No immunostaining for CHOP, HIF-1α or HIF-2α was observed in alveolar or bronchiolar epithelial cells of normal control lung samples (Fig. 2D,H,L and Supplemental Fig. 1D,H) or in the preserved lung remote from pathological area.

### Hypoxia induces CHOP and apoptosis in rat lung

**Figure 3:**
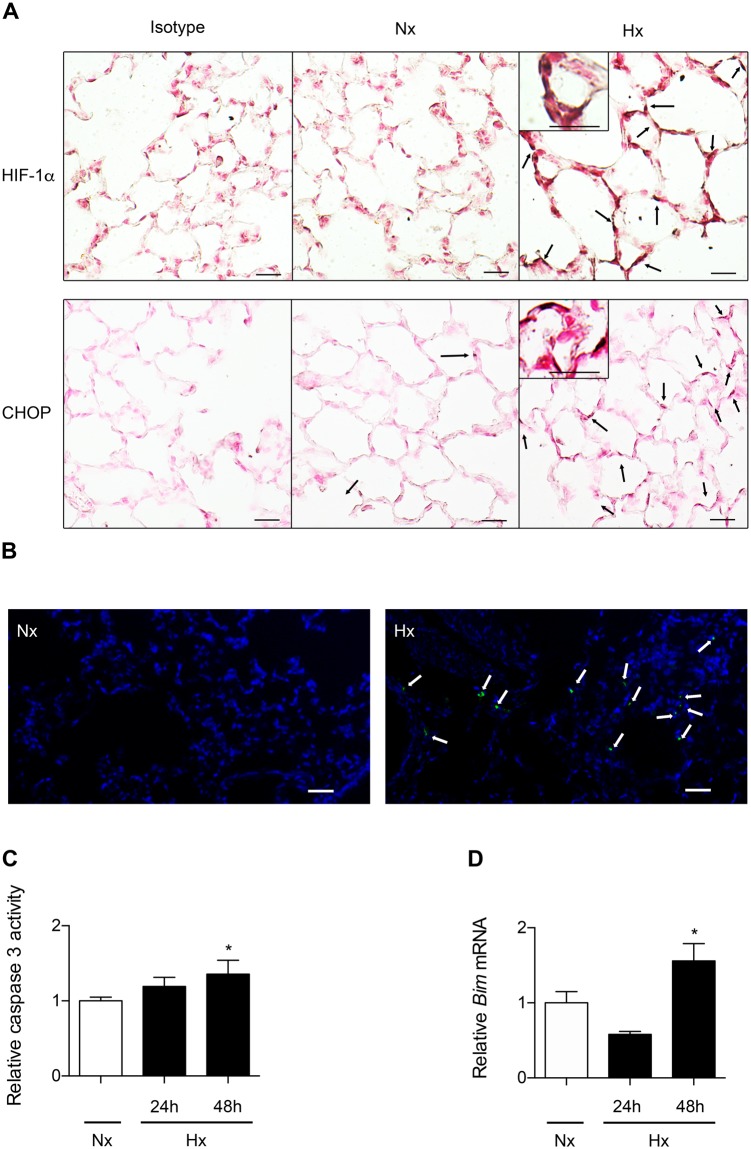
Hypoxia induces HIF-1α, CHOP and apoptosis in rat lung exposed to hypoxia. Lungs of rats stabulated in normoxia (Nx) (21% of O_2_) or exposed to hypoxia (Hx) (8% FiO_2_-like) during 24 h, 48 h or 72 h were used for immunohistochemistry, TUNEL assay, apoptosis enzymatic assay and RT-qPCR analysis. Paraffin-embedded rat lung serial sections were immunostained for HIF-1α or CHOP and counterstained with nuclear fast red. Original magnification: X400 (**A**). Rat lung sections have been TUNEL-labeled (green) (shown by arrow) and DAPI-stained (blue). Original magnification: X200, scale bars represent 100 μm (**B**). The activity of effector caspase 3 was evaluated by enzymatic assay in rat lung homogenates (**C**). *Bim* mRNA expression levels were evaluated in rat lung homogenates by RT-qPCR (**D**). n = 6–8 rats per group. Data were submitted to a Kruskal-Wallis one-way analysis of variance followed by a Dunn’s multiple comparison tests with *P < 0.05 representing a significant difference as compared with normoxic condition.

Immunohistochemistry experiments on rat lung tissues revealed that exposure of rats to hypoxia (equivalent to 8% FIO_2_) for 24 h induced HIF-1α stabilization and CHOP protein expression specifically in the alveolar epithelium (Fig. 3A). The effect of hypoxia on apoptosis was studied by TUNEL assay. As shown in Fig. 2B, the presence of DNA strand breaks was detected in the lungs of rats exposed to hypoxia for 72 h, but not in normoxic rat lungs (58 ± 9 positive cells per field *vs* 0 in hypoxic and normoxic conditions, respectively). Moreover, there was a significant activation of effector caspase 3 (Fig. 3C) and the expression of the pro-apoptotic *Bim* mRNA (Fig. 3D) in lung homogenates of rat exposed 48 h to hypoxia (*P* < 0.05).

### Hypoxia induces CHOP and apoptosis in primary rat alveolar epithelial cells

**Figure 4:**
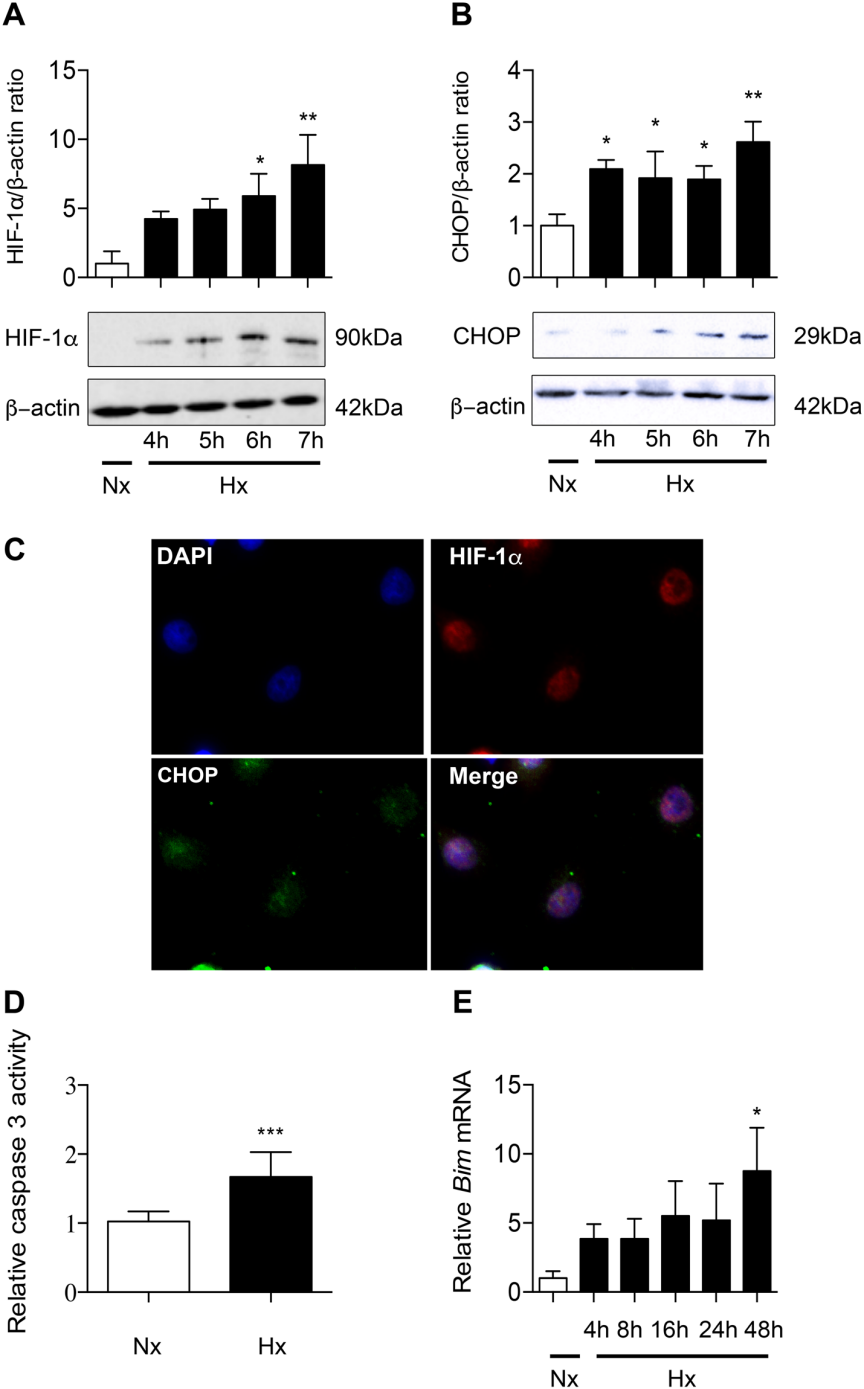
Hypoxia induces HIF-1α, CHOP and apoptosis in alveolar epithelial cells exposed to hypoxia. Primary rat AECs were exposed to normoxia (Nx) (21% of O_2_) or hypoxia (Hx) (1.5% of O_2_) for increasing times (4–24 h). Protein levels of HIF-1α (**A**) and CHOP (**B**) were evaluated by western blotting and were normalized to the corresponding β-actin signal. Rat AECs were exposed to 6 h-hypoxia and immunolabeled for HIF-1α (red) and CHOP (green). DAPI was used to stained nucleus (blue) (**C**). The activity of effector caspase 3 was evaluated by enzymatic assay (**D**). Expression of the pro-apoptotic marker *Bim* was evaluated by RT-qPCR (**E**). n = at least 5 independent AECs cultures. Data were submitted to a Kruskal-Wallis one-way analysis of variance followed by a Dunn’s multiple comparison tests. *P < 0.05, **P < 0.01 and ***P < 0.001 represent a significant difference as compared with normoxic condition.

Primary rat AECs were exposed to 21% O_2_ or 1.5% O_2_ for 4 h to 24 h. HIF-1α protein was detected by western blotting in hypoxic cells after 4 h of exposure and its expression level increased progressively thereafter (Fig. 4A). No signal was detected in normoxic AECs. Similarly, a 2-fold overexpression of CHOP protein was observed in AECs after a 4 h-hypoxic exposure (*P* < 0.001) (Fig. 4B). Immunostaining of HIF-1α and CHOP in AECs exposed to hypoxia for 6 h revealed that these transcription factors were both localized in the nucleus (Fig. 4C). At 24 h of hypoxia, caspase 3 activity was significantly increased (*P* < 0.001) (Fig. 4D). *Bim* mRNA expression levels was markedly upregulated after 24 h of hypoxia (7.56 ± 3.51-fold change as compared with normoxic condition, *P* < 0.05) (Fig. 4E).

### ER stress is involved in hypoxia-induced apoptosis of alveolar epithelial cells through CHOP regulation

#### ER stress is involved in hypoxia-induced apoptosis of alveolar epithelial cells

**Figure 5:**
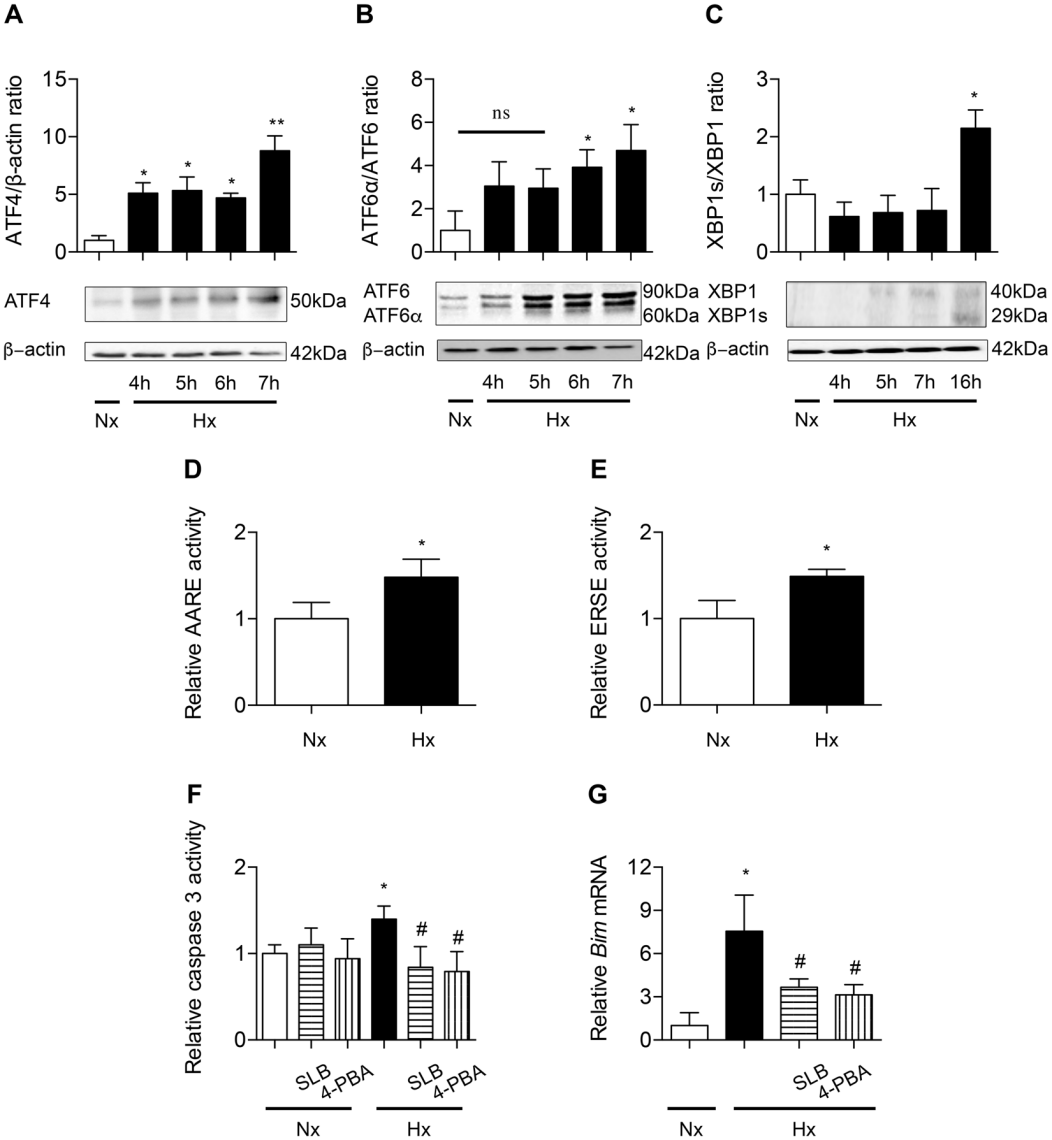
ER stress is involved in hypoxia-induced alveolar epithelial cells apoptosis. Primary rat AECs were placed in normoxia (Nx) (21% of O_2_) or exposed to hypoxia (Hx) (1.5% of O_2_) for increasing times (4–24 h). Protein levels of ATF4 (**A**), ATF6α/ATF6 ratio (**B**) and XBP1s/XBP1 ratio (**C**) were evaluated by western blotting. Quantification has been done on at least 5 independent experiments, representing the densitometry analysis of each proteins of interest reported to β-actin. Primary rat AECs were transfected with plasmids coding for luciferase reporter activity of ATF4 (the amino acid response element: AARE) (**D**) or ATF6α/XBP1s (the endoplasmic reticulum response element: ERSE) (**E**) and exposed to hypoxia for 6 h. Primary rat AECs were placed in normoxia or exposed to hypoxia for 24 h in the presence or absence of ER stress modulators salubrinal (SLB, 100 µM) or 4-phenylbutyrate (4-PBA, 100 mM). The activity of effector caspase 3 was evaluated by enzymatic assay (**F**), and expression of the pro apoptotic marker *Bim* was evaluated by RT-qPCR (**G**). n = at least 5 independent experiments. Data were submitted to a Kruskal-Wallis one-way analysis of variance followed by a Dunn’s multiple comparison tests, except for AARE and ERSE activity data submitted to a Mann-Whitney analysis. *P < 0.05, and **P < 0.01: significantly different from control value in normoxic cells. ^#^*P* < 0.05: significantly different from the value in untreated hypoxic cells. ns: non-significant difference between normoxic condition and hypoxic condition.

As shown in Fig. 4, ATF4 protein level (expressed as the ATF4/β-actin ratio) and ATF6α/ATF6 ratio increased in a time-dependent manner under hypoxic condition. As compared to normoxic condition, a 6 h-hypoxic exposure induced a 8-fold increase in ATF4 and a 5-fold increase in ATF6α/ATF6 ratio (*P* < 0.05) (Fig. 5A,B). The XBP1 spliced form/full-length protein (XBP1s/XBP1) ratio was significantly increased after 16 h hypoxia exposure (2-fold change as compared with normoxic condition, *P* < 0.05) (Fig. 5C). Control experiments showed that these proteins were also induced by tunicamycin (Supplemental data 2). A significant increase in the capacity of both ATF4 and ATF6α/XBP-1s to transactivate their respective consensus responsive elements (AARE and ERSE) upstream the luciferase gene was shown after a 6 h-exposure to hypoxia, as compared with normoxic condition (*P* < 0.05) (Fig. 5D,E). To evaluate the implication of hypoxia-induced ER stress in apoptosis, AECs were treated with salubrinal (SLB) or 4-phenylbutyrate (4-PBA), two ER stress inhibitors^20,21^, before exposure to hypoxia. SLB and 4-PBA had no effect on caspase 3 activity in normoxic cells. As shown in Fig. 5F, the increase in caspase 3 activity in response to a 24 h hypoxic exposure was fully prevented by SLB and 4-PBA treatment. Both SLB and 4-PBA blunted the hypoxia-induced increase in *Bim* expression (*P* < 0.05) (Fig. 5G).

#### CHOP contributes to hypoxia-induced apoptosis of alveolar epithelial cells

**Figure 6:**
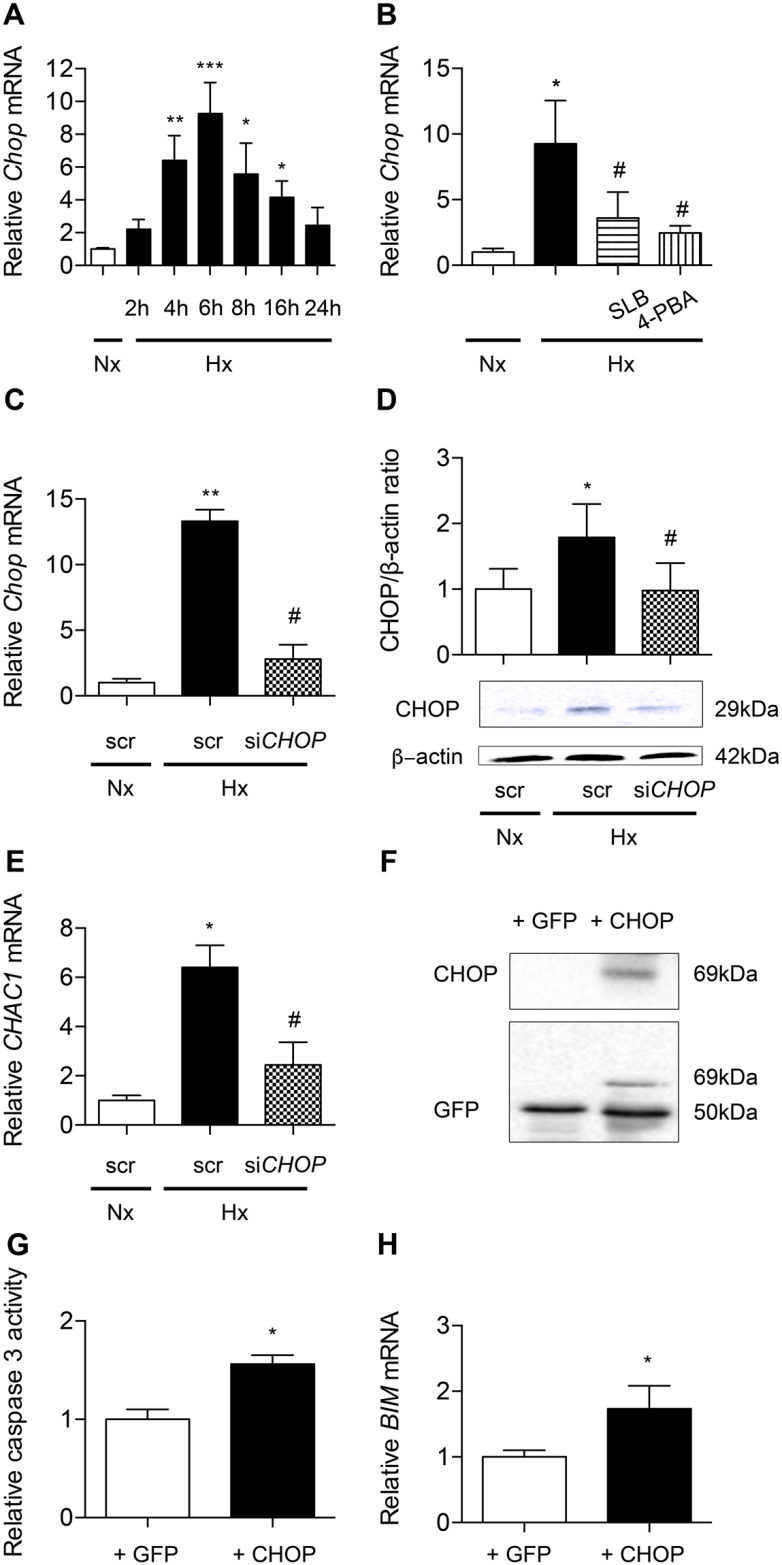
CHOP is involved in hypoxia-induced alveolar epithelial cells apoptosis. *Chop* mRNA expression was evaluated by RT-qPCR in primary rat AECs placed in normoxia (Nx) (21% of O_2_) or exposed to hypoxia (Hx) (1.5% of O_2_) for increasing times (4-24 h) (**A**). *Chop* mRNA expression was evaluated by RT-qPCR in primary rat AECs treated with 100 μM salubrinal (SLB) or 100 mM 4-phenylbutyrate (4-PBA) and exposed 6 h to hypoxia (1.5% of O_2_) (**B**). A549 cells were transfected with *CHOP* siRNA or scrambled (scr) siRNA. 24 h after transfection, A459 cells were placed for 24 h in hypoxia (0.5% of O_2_). CHOP silencing was validated by evaluation of CHOP expression by RT-qPCR (**C**) and western blotting (**D**). Expression of the targeted CHOP pro-apoptotic marker *CHAC-1* was evaluated by RT-qPCR in transfected A549 (**E**). A549 cells were transfected with an empty GFP vector (+GFP) or a plasmid coding for CHOP-GFP protein fusion (+CHOP). Transfection efficiency was evaluated by western blotting (**F**). Activity of effector caspase 3 (**G**) and *BIM* mRNA expression (**H**) were evaluated after 24 h exposure to hypoxia (0.5% of O_2_). n = at least 5 independent experiments. Data were submitted to a Kruskal-Wallis one-way analysis of variance followed by a Dunn’s multiple comparison tests, except for caspase 3 activity and *BIM* mRNA expression data submitted to a Mann-Whitney analysis. *P < 0.05, **P < 0.01 and ***P < 0.001: significantly different from normoxic control value (**A**,**B**), from value in normoxic cells transfected with scrambled si-RNA (**C**–**E**) or from GFP-transfected cells value (**G**,**H**). ^#^*P* < 0.05 significantly different from value in untreated hypoxic cells (B) or from value in hypoxic cells transfected with scrambled siRNA (**C**–**E**).

As shown in Fig. 6A, *Chop* mRNA transcripts markedly increased under hypoxic condition with a peak corresponding to a 9-fold increase at 6 h of exposure, as compared with normoxic condition (*P* < 0.001). Interestingly, treatment of hypoxic AECs with SLB and 4-PBA prevented *Chop* mRNA upregulation (Fig. 6B) (P < 0.05). The silencing of *CHOP* was then achieved in A549. After verifying the efficiency of *CHOP* silencing on the mRNA and protein expression under hypoxic condition (Fig. 6C,D), we tested its impact on hypoxia-induced markers of apoptosis. *CHOP* silencing markedly blunted the hypoxia-induced increase in mRNA transcript levels of *CHAC1*, a CHOP-regulated pro-apoptotic gene (Fig. 6E). In a complementary experiment, CHOP was overexpressed in A549 cells by transfection of a CHOP-GFP protein expressing plasmid (Fig. 6F). Control has been made with the backbone GFP vector. Caspase 3 activity significantly increased in normoxic cells overexpressing CHOP as compared with the value observed in cells transfected with the GFP empty vector used as control (*P* < 0.05) (Fig. 6G). The same effect was observed for *BIM* mRNA (*P* < 0.05) (Fig. 6H).

### HIF-1α upregulates UPR pathways and CHOP in alveolar epithelial cells

**Figure 7:**
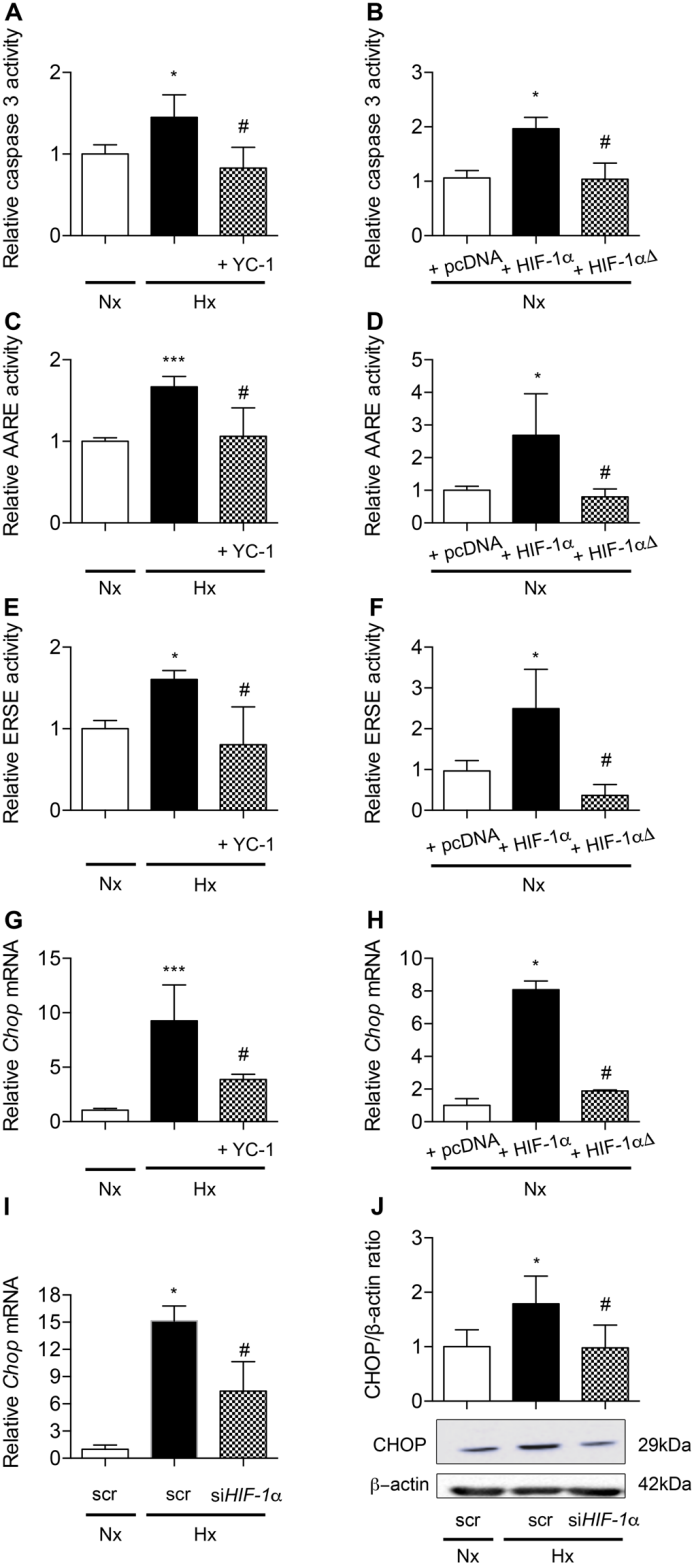
HIF-1α is involved in ER-stress induced CHOP-dependent apoptosis in alveolar epithelial cells. Caspase 3 activity was evaluated in primary rat AECs treated or not with the HIF-1α inhibitor YC-1 (10 μM) and exposed to normoxia (Nx) (21% of O_2_) or hypoxia (Hx) (1.5% of O_2_) for 24 h (**A**). A549 cells were transfected with either an empty pcDNA3.1 vector or a plasmid encoding HIF-1α or a mutated HIF-1α (HIF-1αΔ) unable to transactivate. Caspase 3 activity was measured 48 h after transfection (**B**). ATF4 (**C**) or ATF6α/XBP1s (**E**) relative transcriptional activities were evaluated in primary rat AECs treated or not with YC-1 and exposed to normoxia or hypoxia for 6 h. In A459 cells co-transfected with either an empty pcDNA3.1 vector or a plasmid encoding HIF-1α or a mutated HIF-1αΔ, ATF4 (**D**) or ATF6α/XBP1s (**F**) relative transcriptional activities were measured 48 h after transfection. *CHOP* mRNA expression was evaluated by RT-qPCR in primary rat AECs treated or not with YC-1 and exposed to normoxia or hypoxia for 6 h (**G**). *CHOP* mRNA expression was evaluated by RT-qPCR in A459 cells transfected with either an empty pcDNA3.1 vector or a plasmid encoding HIF-1α or mutated HIF-1αΔ 48 h post-transfection (**H**). A549 cells were transfected with *HIF-1α* siRNA or scrambled (scr) siRNA, and exposed to hypoxia (0.5% of O_2_) for 24 h. CHOP expression was evaluated by RT-qPCR (**I**) and western blotting (**J**). n = at least 5 experiments. Data were submitted to a Kruskal-Wallis one-way analysis of variance followed by a Dunn’s multiple comparison tests. *P < 0.05, ***P < 0.001: significantly different from normoxic control value (**A**,**C**,**E**,**G**), from value in normoxic scrambled-transfected cells (**I**–**J**) or from value in normoxic pcDNA3.1-transfected cells (**B**,**D**,**F**,**H**). ^#^*P* < 0.05, significantly different from value in untreated hypoxic cells (**A**,**C**,**E**,**G**), in hypoxic cells transfected with scrambled siRNA (**I**–**J**), or in hypoxic cells transfected with pcDNA3.1 (**B**,**D**,**F**,**H**).

We then documented the implication of HIF-1α in the induction of apoptosis and UPR pathways regulation. First, we verified the efficiency of HIF-1α pharmacological inhibitor YC-1 on the transcriptional activity of HIF-1α (Supplemental data 3A). Second, we confirmed the capacity of A549 cells transfected with a plasmid coding for HIF-1α to transactivate a minimal promoter containing HRE specific consensus upstream the luciferase gene (Supplemental data 3B). In Fig. 7A, we show that the increase in caspase 3 activity observed in hypoxic condition was completely abolished by YC-1. Interestingly, a significant increase in caspase 3 activity was observed in A549 cells overexpressing HIF-1α, as compared to control condition, i.e. cells transfected with an pcDNA 3.1 empty vector (*P* < 0.05) (Fig. 7B). No significant change was observed with HIF-1αΔ, a HIF-1α mutant unable to transactivate^22^.

In primary rat cells exposed to hypoxia, YC-1 treatment completely abolished the increase of the luciferase activity obtained after fixation of either ATF4 or ATF6α/XBP1s on their minimal promoter containing AARE or ERSE specific consensus respectively (Fig. 7C,E). Moreover, the co-transfection of A549 cells with a plasmid encoding HIF-1α in combination with the ATF4 responsive element or the ATF6α/XBP1s responsive element upstream the luciferase gene resulted in a more than 2-fold increase in the luciferase activity compared to control condition (*P* < 0.05) (Fig. 7D,F). No significant change was observed with the inactive mutant HIF-1αΔ.

Incubation of primary rat AECs with YC-1 significantly blunted hypoxia-induced upregulation of *Chop* mRNA expression (*P* < 0.05) (Fig. 7G). Conversely, transfection of normoxic A549 with HIF-1α induced an 8-fold increase in *Chop* mRNA expression as compared with control (Fig. 7H). No significant change was observed with the inactive mutant HIF-1αΔ. After we verified the efficiency of *HIF-1α* silencing in A549 cells exposed to hypoxia for 24 h (Supplemental data 4), we demonstrated that *HIF-1α* siRNA significantly reduced *CHOP* mRNA levels (*P* < 0.05) (Fig. 7I), as well as CHOP protein levels (*P* < 0.05) (Fig. 7J) in response to hypoxia.

## Discussion

Expression of ER stress markers has been evidenced in hyperplastic AECs from pulmonary biopsies of sporadic IPF patients^7^, but the trigger(s) for epithelial ER stress in this context is (are) not clearly identified. Considering the fact that HIF-1α protein is specifically expressed in AECs from IPF lungs or bleomycin-treated mouse lungs^19,23^, we hypothesized that localized alveolar hypoxia and/or HIF-1α may play a role in this process. The aim of the present study was therefore to investigate the potential involvement of hypoxia and/or HIF-1α in the modulation of ER stress and the subsequent pro-fibrotic features of AECs in the context of pulmonary fibrosis^5,9^. Our results provide evidence that a hypoxic microenvironment and the stabilization of HIF-1α induce ER stress in AECs, the expression of CHOP, a pro-apoptotic factor currently used as a marker of ER stress in IPF^7^, and subsequent apoptosis.

In the present study, analysis of lung serial sections of bleomycin- induced pulmonary fibrosis in mice as well as UIP areas in lung biopsies undertaken for IPF diagnosis evidenced the co-expression of HIF-1α and CHOP in hyperplastic AECs covering the remodeled parenchymal air spaces. HIF-1α and CHOP were not detected in the preserved lung at distance from pathological areas, as in normal control lungs. This observation is in agreement with two previous studies showing that the expression of HIF-1α is mainly restricted to AECs located within fibrotic areas in IPF lungs^19,23^. Because HIF-1α protein is typically stabilized in case of oxygen deprivation, the expression of HIF-1α observed in IPF lung could likely be due to localized alveolar hypoxia in remodeled parenchymal airspaces with compromised ventilation. In line with this hypothesis, *Burman et al*. recently proposed that localized hypoxia worsens pulmonary fibrosis and could contribute to CHOP overexpression in IPF lungs^24^. However, the link between HIF-1 expression and the expression of ER stress markers and CHOP remained unclear^24^. Alveolar hypoxia may also certainly be encountered during acute exacerbations of IPF. To the best of our knowledge, the presence of localized alveolar hypoxia has never been directly proven in human IPF, probably due to technical limitations. By contrast, experimentally, Weng *et al*. as Burman *et al*. demonstrated the presence of localized hypoxia (together with epithelial HIF-1α expression) in fibrotic lung tissues from mice treated with bleomycin using the hypoxyprobe staining technique^19,23,24^. However, it cannot be excluded that HIF-1α expression in IPF lungs might be due in some cases to non-hypoxic triggers, as previously described in other pathological conditions^25^.

Whatever the cause of HIF-1α stabilization in IPF lung, our immunostaining data suggest a link between HIF-1α expression and ER stress, inasmuch as HIF-1α-positive AECs appear to co-express the ER-stress marker CHOP. Supporting this hypothesis, we also observed in AECs from rats exposed to hypoxia the expression of both transcription factors, HIF-1α and CHOP, as well as the induction of apoptosis as assessed by TUNEL and caspase 3 activation assays, and the expression of the pro-apoptotic marker *Bim*. *In vitro* experiments confirmed the *in vivo* results. First, exposure of primary rat AECs to hypoxia led to a time-dependent induction of HIF-1α and CHOP expression, and both transcription factors were localized in the nucleus. Also, hypoxia induced caspase 3 activation and an increase in mRNA transcripts levels encoding *Bim*, as described *in vivo*. Next, our data strongly suggest that hypoxia-induced apoptosis was, at least in part, related to the induction of the UPR pathways. The three branches of the UPR pathways, ATF4, ATF6α and XBP-1s, were clearly activated in primary rat AECs as a result of exposure to hypoxia. Indeed, treatment of AECs with 4-phenylbutyrate (4-PBA), a FDA-approved drug for the treatment of urea cycle disorder used as a chemical chaperone improving protein misfolding^21^, markedly prevented the hypoxia-induced increase in caspase 3 activity and *Bim* expression. Interestingly, other studies have also used this drug to attenuate the ER stress observed after injury resulting from a hypoxic environment in various situations, *ie* against hypoxia-induced pulmonary hypertension, hypoxia-induced cardiovascular damage^26^ and more interestingly, EMT during bleomycin-induced fibrosis^27^. Salubrinal (SLB), considered as a selective modulator of the ATF4 pathway during ER stress-mediated apoptosis^28^, had the same effect as 4-PBA, highlighting the involvement of the UPR/ATF4 pathway in the induction of apoptosis in hypoxic AECs.

Our data also provide evidence that the transcription factor CHOP plays a critical role in hypoxia-induced apoptosis of AECs. CHOP, a target of ATF4 and ATF6 UPR pathways, is considered as one of the most important mediators of ER stress-induced apoptosis^29^. One well-accepted mechanism proposed for the pro-apoptotic role of CHOP is that it can interact with transcriptional repressors, inhibiting the transcription of the anti-apoptotic *Bcl2* gene^12^, and co-activating the transcription of the pro-apoptotic factor *Bim*^13^. CHOP was also shown to induce the pro-apoptotic factor *Chac-1*, the overexpression of which strongly induces apoptosis^14^. Here, we observed that the upregulation of CHOP induced by hypoxia in AECs was markedly blunted by the use of ER stress inhibitors, 4-PBA and SLB. In AECs exposed to normoxia, overexpression of CHOP induced caspase 3 activity and *BIM* expression. In AECs exposed to hypoxia, *CHOP* gene silencing clearly decreased *CHAC1* mRNA levels. Although these findings strongly suggest a major role of CHOP in hypoxia-induced apoptosis, we cannot exclude the involvement of additional pro-apoptotic pathways as JNK/AP-1 which transcriptional activity is known to participate in ER stress induced-cell apoptosis^30^.

Finally, we investigated the specific role of HIF-1α in hypoxia-induced ER stress and CHOP-mediated apoptosis in AECs, and whether HIF-1α would be able *per se* to induce ER stress and CHOP-mediated apoptosis, independently of a hypoxic context. Hypoxia-induced apoptosis has been previously addressed in AECs and a role for the HIF pathway has been evoked^31,32^. Specifically, we and other groups demonstrated *in vitro* the activation of the Bcl-2 family member Bnip3, a pro-apoptotic target of HIF-1α, in hypoxic AECs^32,33^. Our data confirm the major role of HIF signaling pathway in hypoxia-induced apoptosis, as over-expression of HIF-1α was able to induce caspase 3 activity and *Bim* in normoxic AECs, whereas its pharmacological inhibition by YC-1 abolished the increase in caspase 3 activity in hypoxic AECs. We also provide evidence that HIF-1α, in addition to its effect on Bnip3, can also induce apoptosis in AECs through the induction of UPR pathways and the up-regulation of CHOP. Our results show that overexpression of HIF-1α increased ATF4 and ATF6α/XBP1s transcriptional activities in normoxic AECs, as well as the expression levels of *Chop* mRNA transcripts. Also, pharmacological inhibition of HIF-1α in hypoxic AECs completely abolished the hypoxia-induced increase in ATF4 and ATF6α/XBP1s transcriptional activities. Finally, pharmacological inhibition of HIF-1α or HIF-1α gene silencing both markedly reduced the hypoxia-induced increase in *Chop* mRNA levels. It is well-known that CHOP is transcriptionally activated by the three pathways of the UPR signaling^11^. Therefore, the upregulation of CHOP induced by HIF-1α in AECs may likely be indirect, *i.e*. due to the upregulation of ATF4 and ATF6α/XBP1s transcriptional activities. In a recently published study conducted on HIF1/2^−/−^ bleomycin-treated mice exposed to hypoxia, no modification of CHOP expression was observed, and the authors proposed that the regulation of CHOP expression is independent of HIF^24^. However, as the effects of HIF deletion and/or hypoxic exposure on either ATF4 or ATF6/XBP1s expression were not documented in this latter study the question on the critical role of HIF-1 on CHOP regulation remained open. It is noteworthy that CHOP could also be a direct target of HIF-1α, inasmuch as analysis of the CHOP promoter revealed the presence of at least 3 specific Hypoxic Response Elements sequences (−89/−93 pb, −330/−334 pb and −336/−340 pb upstream the transcription start site sequence)^34^. It is interesting to note that the stimulatory effects of HIF-1α on UPR pathways and CHOP expression we observed in AECs are relatively cell-specific and not necessarily reproduced in other cell types. For instance, in β-pancreatic cells, the pro-apoptotic effect of hypoxia and the hypoxic activation of the UPR signaling pathways appeared to be independent of HIF-1α^35^. By contrast, in hepatocytes or in embryonic fibroblasts, inhibition of HIF-1α was shown to exacerbate lipoapoptosis and to dramatically induce CHOP expression, as a consequence of the loss of HIF-1α repressive activity on the CHOP promoter^36^. These discrepancies demonstrating sometimes an activator or an inhibitor role for HIF-1α on CHOP regulation highlight the cell type specificity of this relationship.

In conclusion, the present study demonstrates that hypoxia and HIF-1α *per se* induce UPR pathways and CHOP-mediated apoptosis in AECs. Our results suggest that the localized alveolar hypoxia or at least the stabilization of HIF-1α in AECs could trigger ER stress and related cell damages, thus contributing to the development of lung fibrosis. Strategies targeting the HIF/UPR/CHOP pathway could potentially represent a new therapeutic issue to limit the development of pulmonary fibrosis.

## Materials and Methods

### Statement

All experiments and methods were performed in accordance with relevant guidelines and regulations. Methods for collecting and handling human samples were approved by the local ethical committee (CLEA-2017-040). The requirement for signed informed consent was waived according to the French legislation. Model of rat exposed to acute hypoxia and AECs isolation experiments were approved by the ethical committee (C2EA-06, C9300801, APAFIS #7846 and C9300801, APAFIS #8150 respectively) and done in accordance with the European Communities Council for animal.

### Human lung tissue samples

Formalin-fixed and paraffin embedded tissue samples were obtained from pulmonary biopsies of 3 IPF patients and normal lung areas in non IPF patients considered as controls (details in Table 1).

**Table 1:** Patient clinical data.

	IPF patient 1	IPF patient 2	IPF patient 3	Control patient
Age	68	57	59	70
Gender	Female	Male	Male	Male
Smoking status* (Pack Year)	10	10	30	32
Pulmonary Function Tests	FVC 58%	FVC 73%	FVC 68%	FEV1 96%
	DLCO 40%	DLCO 72%	DLCO 48%	
Histology	Usual interstitial pneumonia	Usual interstitial pneumonia	Usual interstitial pneumonia	Lung parenchyma remote from lung adenocarcinoma

All patients were ever-smokers.

Abbreviations: FVC, Forced Vital Capacity; DLCO, Diffusion capacity of the Lung for Carbon monoxide; FEV1, Forced Expiratory Volume in one second.

### Animal models

#### Bleomycin-induced lung fibrosis in mice

A single intra-tracheal injection of bleomycin (3, 5 U/g body weight in 100 µl saline) was performed on 8-weeks-old male C57BL/6J as previously described^37^. Experiments were approved by our ethical committee (C2EA-06, C9300801 APAFIS #1258).

#### Model of rat exposed to acute hypoxia

4-week old male Sprague-Dawley rats (n = 6–8 per group) were exposed for 16, 24, 48 or 72 h to hypobaric hypoxia in a Plexiglas chamber maintained at a pressure of 328 mmHg (simulating a 8% FIO_2_).

### Rat alveolar epithelial cell isolation and culture

AECs were isolated from 4-week old male Sprague-Dawley rats according to a procedure previously described^38^. Isolated cells consisted of 92% of ATII cells, and cell viability was 95%^38^. Cells were cultured in DMEM containing 25 mM D-glucose, 10 mM Hepes, 23.8 mM NaHCO_3_, 2 mM L-glutamine, 10% foetal bovine serum (FBS), 50 U/ml penicillin, 50 µg/ml streptomycin, 10 µg/ml gentamycin, 10 µg/ml amphotericin B (Thermo Scientific) and placed at 37 °C with 5% CO_2_ in a humidified incubator. The human alveolar epithelial A549 cell line (ATCC) was used for gene silencing experiments.

### Hypoxic exposure and drug treatments of alveolar epithelial cells

Three days after isolation, primary rat AECs were exposed to 1.5% O_2_ (equivalent to a 45 mmHg oxygen tension in cell medium). 100 µM salubrinal (Santa Cruz) or 100 mM 4-phenylbutyrate (4-PBA) (Sigma) were used to inhibit UPR pathways. 10 μM of YC1 (Sigma) was used to inhibit HIF-1α expression. A549 cells were exposed to 0.5% O_2_ to obtain the same pattern as primary cells regarding UPR and apoptotic response to hypoxia^39,40^.

### Transient transfection of alveolar epithelial cells and luciferase assays

For each transcription factor, a plasmid coding for a firefly luciferase gene downstream tandem repetition of its specific responsive elements on the promoter region has been co-transfected with a pRL-SV40 plasmid (Promega). pRL-SV40 plasmid expressed renilla reniformis luciferase (RL) downstream the SV40 promoter and was used to normalize the luciferase response to the efficiency of the transfection. Primary rat AECs were transfected with plasmids containing the hypoxia response element (HRE) for the specific binding of HIF, the amino acid response element (AARE) for ATF4 or the endoplasmic reticulum stress response element (ERSE) for the binding of both ATF6α and XBP1s, all cloned upstream the luciferase reporter gene. The plasmid encoding HIF-1α (#181949, Addgene) was used to decipher the role of HIF-1α in UPR pathways and CHOP induction. The plasmid encoding CHOP-GFP (#21898, Addgene) was used to decipher the role of CHOP in the induction of apoptosis. The empty plasmids pcDNA 3.1 (Invitrogen) or GFP (#632370, Clontech) were used as control.

AECs were transiently transfected with the NEON™ transfection system (Life Technologies) allowing a 25–30% transfection efficiency as previously described^41^. The efficiency of transfection was controlled by western blot evaluating the expression of the transfected genes.

### Gene silencing in A549 cell line

As transfection efficiency in primary rat AECs is low (less that 30% of cells expressing the transgene), we used A549 for gene silencing experiments. A549 cells were transfected with *CHOP* siRNA sequences 5′AAGAACAGCAGAGGUCACAA-ttt3′, 5′GCCUGGUAUGAGGACCUGC-ttt3′ or with *HIF-1α* siRNA sequence 5′CUGAUGACCAGCAACUUGA-ttt3′ using Lipofectamine® 2000 according to the manufacturer’s instructions (ThermoFisher). Transfection efficiency reached 80%.

### Western blot analyses

Total proteins from rat primary AECs and A549 cells exposed to normoxia or hypoxia were extracted as previously described^38^ and probed with the appropriate antibodies (Table 2). Protein ratios or expression levels were normalized to the corresponding expression level of anti-β actin, used as a loading control.

**Table 2:** Antibodies used for western blot experiment.

Antibodies (anti-)	Immunogen	Dilutions	Manufacturer’s reference
ATF4 (polyclonal rabbit)	C-ter human ATF4, cross-react with human, mouse, rat	1/1000^e^	sc-200 (Santa Cruz Biotechnology)
ATF6α (polyclonal rabbit)	Fused protein including residues 31–310 of human ATF6, cross-react with human, mouse, rat	1/1000^e^	sc-22799 (Santa Cruz Biotechnology)
XBP1 (polyclonal rabbit)	N-ter human XBP1, cross-react with human, mouse, rat	1/1000^e^	A37152 (Sigma Aldrich)
CHOP (mouse monoclonal)	Full length mouse CHOP, cross-react with human, mouse, rat	1/1000^e^	NB600–1335 (Novus)
HIF-1α (rabbit polyclonal)	Fused protein including residues 530–825 of mouse HIF-1α, cross-rabbit with human, mouse, rat	1/1000^e^	NB 100–479 (Novus)
β-ACTIN (rabbit polyclonal)	Synthetic actin N-ter, cross-react with mouse, human, rat	1/5000^e^	A2103 (Sigma Aldrich)

### RNA extraction and reverse transcriptase-polymerase chain reaction

Total RNA was extracted from rat AECs and A549 cells exposed to normoxia or hypoxia. Primers were designed to have a 25–30 cycle threshold values (Table 3).

**Table 3:** Primers used for Real-Time Polymerase Chain Reaction.

Gene	Forward Primer	Reverse primer
**Rat**		
*Chop*	TGTTGAAGATGAGCGGGTGG	TGGACCGGTTTCTGCTTTCA
*Bim*	TTACACGAGGAGGGCGTTTG	CCAGACCAGACGGAAGATGA
*β-Actin*	ACCGTGAAAAGATGACCCAGA	CACAGCCTGGATGGCTACGT
**Human**		
*CHOP*	TTCTCTGGCTTGGCTGACTG	CTGCGTATGTGGGATTGAGG
*CHAC1*	CCTGAAGTACCTGAATGTGCGAGA	GCAGCAAGTATTCAAGGTTGTGGC
*β-ACTIN*	AGAGCTACGAGCTGCCTGAC	AAAGCCATGCCAATCTCATC

### Apoptotic pathways activation assay

Caspase-Glo 3/7® assay (Promega) was used in lung homogenates, rat primary AECs exposed to hypoxia and in A549 cells transfected with plasmid coding for GFP or CHOP-GFP protein (#21898 Addgene) to evaluate apoptosis. Briefly, 50 µl of reagent containing a proluminescent caspase substrate was added to 5 µg of cell lysates. The cleavage of the substrate by the caspase 3 present within the samples liberates free aminoluciferin, which is consumed by the luciferase, generating a “glow-type” luminescent signal that is proportional to caspase 3 activity.

### Lung immunohistochemistry and cell immunofluorescence

Sections of paraffin-embedded bleomycin-treated mice and rat lung samples as lung biopsies from IPF and controls were incubated overnight at 4 °C with anti-CHOP, anti-HIF-1α, or anti-HIF-2α antibodies or with corresponding isotypes (Table 4). AECs cultured 6 h in hypoxic condition (1.5% O_2_) were immunostained for HIF-1α and CHOP. After fixation with 4% paraformaldehyde and cell membrane permeabilization with Triton X100, cells were incubated overnight with anti-CHOP and anti-HIF-1α antibodies (see Table 4). Next day, after three washes, AECs were incubated with Alexafluor™ secondary antibodies solution. Nucleus was labelled with DAPI.

**Table 4:** Antibodies used for immunohistochemistry and immunofluorescence experiments.

Antibodies (anti-)	Immunogen	Dilutions	Manufacturer’s reference
**Human**			
HIF-1α (rabbit polyclonal)	Fused protein including residues 530–825 of mouse HIF-1α, cross-react with human, mouse, rat	1/250^e^	NB100-479 (Novus)
HIF-2α (rabbit polyclonal)	C-Term of mouse/human HIF-2α protein	1/250^e^	NB 100-132 (Novus)
CHOP (mouse monoclonal)	Full length mouse CHOP, cross-react with human, mouse, rat	1/100^e^	NB600-1335 (Novus)
**Rat**			
HIF-1α (rabbit polyclonal)	Fused protein including residues 530-825 of mouse HIF-1α	1/400^e^	NB100-479 (Novus)
CHOP (rabbit polyclonal)	Full length mouse CHOP	1/250^e^	sc-575 (Santa Cruz Biotechnology)

### Statistical analyses

Results were presented as means ± SD. To evaluate differences between groups, all raw data were submitted to a Mann-Whitney and Kruskal-Wallis One-way analysis of variance followed by a Dunn’s multiple comparison tests in PRISM software (version 6, GraphPad). Graphics were performed by PRISM software. A *P* value < 0.05 was considered significant.

## Electronic supplementary material

Supplementary information
