# Supplementary material for: HIF-1α triggers ER stress and CHOP-mediated apoptosis in alveolar epithelial cells, a key event in pulmonary fibrosis
Source: Sci Rep. 2018 Dec 18;8:17939. doi: 10.1038/s41598-018-36063-2 (PMC6299072; doi:10.1038/s41598-018-36063-2)
Supplement: Supplementary file 1 — Supplementary information [file 41598_2018_36063_MOESM1_ESM.pdf]

HIF-1α triggers ER stress and CHOP-mediated apoptosis in alveolar epithelial cells, a key event in pulmonary fibrosis.

Eva Delbrel[1], Abdoulaye Soumare[1], Adnan Naguez[1], Rabab Label[1], Olivier Bernard[1], Alain Bruhat[3], Pierre Fafournoux[3], Geoffrey Tremblais[1], Dominique Marchant[1], Thomas Gille[1,2], Jean-François Bernaudin[1,2,4], Patrice Callard[4], Marianne Kambouchner[2], Emmanuel Martinod[1,2], Dominique Valeyre[1,2], Yurdagül Uzunhan[1,2], Carole Planès[1,2], Emilie Boncoeur[1]

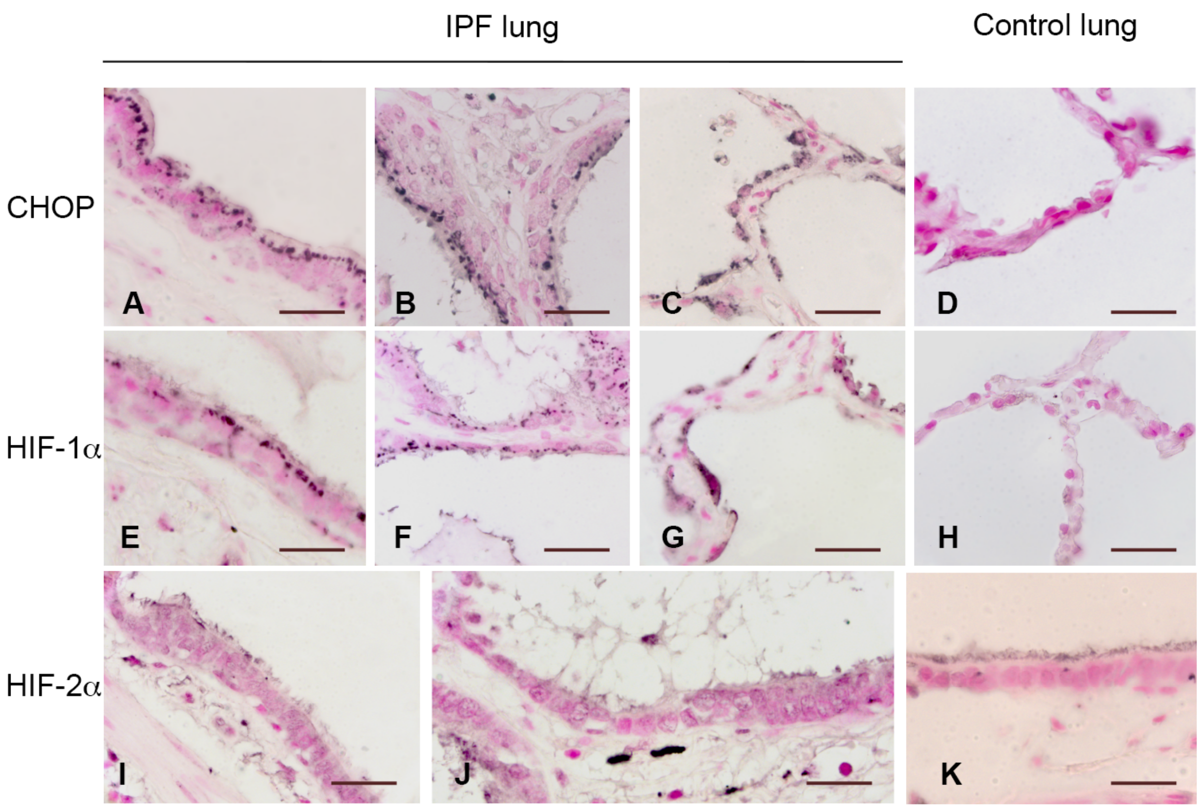

***Supplementary figure 1*.** **Co-expression of CHOP and HIF-1α in alveolar epithelial cells of patients with IPF.** CHOP and HIF-1α expression in alveolar epithelial cells in UIP areas of patients with IPF (UIP) (A-C, E-G), and in normal lung sample (D, H, K). Supranuclear localization of CHOP (A, B) and HIF-1α (E-F) in hyperplastic cells and in pneumocytes in a less fibrotic region (C and G respectively). No labelling was observed for isotype control (L), nor HIF-2α except a faint nonspecific extracellular one above the ciliated cells in a bronchiolization area (I, J). No labelling was observed for CHOP (D) or HIF-1α (H) in the alveolar cells in normal lung (D, H). Sections were counterstained with Nuclear Fast Red. Original magnification: x400. Scale bars represent 200 µm.

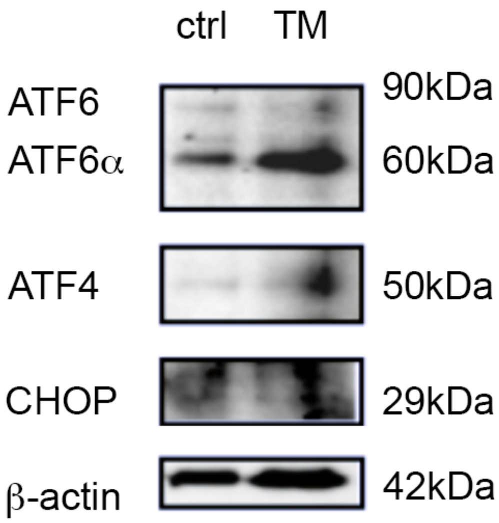

***Supplementary figure 2*. Tunicamycin induces UPR pathways activation in AECs.** Primary rat AECs were treated with the ER stress inducer tunicamycin (TM, 1 μg/ml) or with vehicle (ctrl) for 24 h, and protein levels of ATF6, ATF6α, ATF4, and CHOP were evaluated by western blotting. Representative blot from at least n = 3 independent experiments is shown.

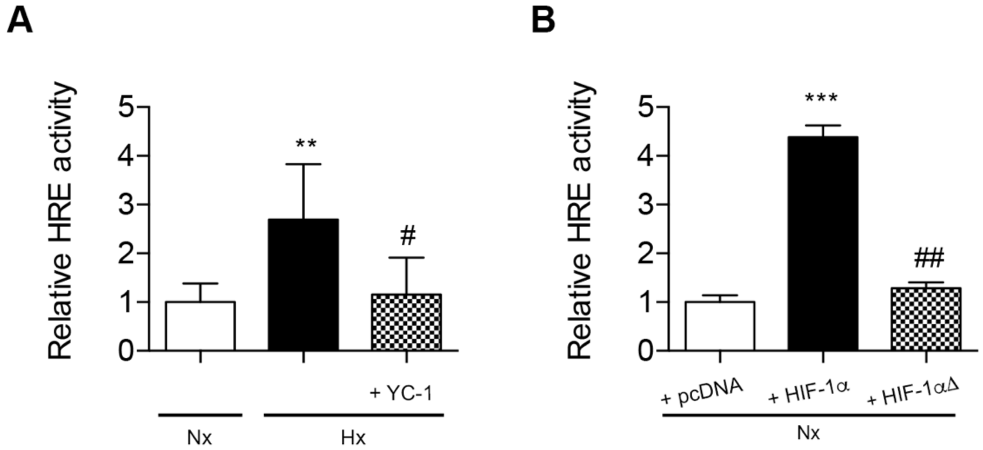

***Supplementary figure 3*. Modulation of HIF-1α transactivation activity by YC-1 treatment and by HIF-1α overexpression.** Primary rat AECs transfected with a plasmid encoding for luciferase reporter activity of hypoxia response element (HRE) and treated or not with the HIF-1α inhibitor YC-1 (10 μM) were exposed to normoxia (Nx) (21% of O_2) or hypoxia (Hx) (1.5% of O_2) for 24 h (A). A549 were co-transfected with either an empty pcDNA3.1 vector or a plasmid encoding HIF-1α or a mutated HIF-1α (HIF-1αΔ), and with a plasmid coding for

luciferase reporter activity of HRE (B). HRE relative transcriptional activity was evaluated 48 h post-transfection. n = at least 6 experiments. Data were submitted to a Kruskal-Wallis one-way analysis of variance followed by a Dunn's multiple comparison tests. **: $P<0.01$ and ***: $P<0.001$: significantly different from control value in normoxic cells (untreated or transfected with pcDNA). #: $P<0.05$ and ##: $P<0.01$: significantly different from values in untreated hypoxic cells or in hypoxic cells transfected with pcDNA3.1 plasmid.

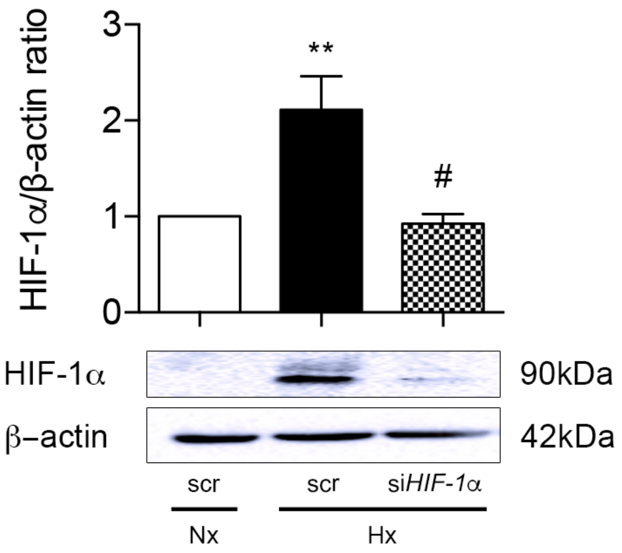

Supplementary figure 4. **Extinction of HIF-1α expression by siRNA.** A549 cells were transfected with scrambled (scr) or *HIF-1α* siRNA. 24 h post-transfection, A459 cells were placed 24 h hypoxia (0.5% of O_2). HIF-1α protein levels were evaluated by western blot 48 h post-transfection. n = 3 experiments, data were submitted to a Kruskal-Wallis one-way analysis of variance followed by a Dunn's multiple comparison tests. **: $P<0.01$: significantly different from normoxic condition and #: $P<0.05$: significantly different as compared with hypoxic cells transfected with scrambled siRNA.
